# A genomic view of experimental intraspecies and interspecies transformation of a rifampicin-resistance allele into *Neisseria meningitidis*

**DOI:** 10.1099/mgen.0.000222

**Published:** 2018-09-25

**Authors:** Kristian Alfsnes, Stephan A. Frye, Jens Eriksson, Vegard Eldholm, Ola Brønstad Brynildsrud, Jon Bohlin, Odile B. Harrison, Derek W. Hood, Martin C. J. Maiden, Tone Tønjum, Ole Herman Ambur

**Affiliations:** ^1^​ Nasjonalt Folkehelseinstitutt, Oslo, Norway; ^2^​ Department of Microbiology, Oslo University Hospital (Rikshospitalet), Oslo, Norway; ^3^​ Department of Molecular Biology, Domain of Infection Control and Environmental Health, Norwegian Institute of Public Health, Oslo, Norway; ^4^​ Department of Methodology Research and Analysis, Domain of Infection Control and Environmental Health, Norwegian Institute of Public Health, Oslo, Norway; ^5^​ The Peter Medawar Building for Pathogen Research, University of Oxford, Oxford, UK; ^6^​ Nuffield Department of Clinical Medicine, University of Oxford, John Radcliffe Hospital, Headington, Oxford, UK; ^7^​ Department of Microbiology, University of Oslo, Oslo, Norway; ^8^​ OsloMet – Oslo Metropolitan University, Oslo, Norway

**Keywords:** *Neisseria meningitidis*, *Neisseria lactamica*, experimental evolution, transformation, homologous recombination, genome sequencing

## Abstract

The spread of antibiotic resistance within and between different bacterial populations is a major health problem on a global scale. The identification of genetic transformation in genomic data from *Neisseria meningitidis*, the meningococcus (Mc), and other bacteria is problematic, since similar or even identical alleles may be involved. A particular challenge in naturally transformable bacteria generally is to distinguish between common ancestry and true recombined sites in sampled genome sequences. Furthermore, the identification of recombination following experimental transformation of homologous alleles requires identifiable differences between donor and recipient, which in itself influences the propensity for homologous recombination (HR). This study identifies the distribution of HR events following intraspecies and interspecies Mc transformations of *rpoB* alleles encoding rifampicin resistance by whole-genome DNA sequencing and single nucleotide variant analysis. The HR events analysed were confined to the genomic region surrounding the single nucleotide genetic marker used for selection. An exponential length distribution of these recombined events was found, ranging from a few nucleotides to about 72 kb stretches. The lengths of imported sequences were on average found to be longer following experimental transformation of the recipient with genomic DNA from an intraspecies versus an interspecies donor (*P*<0.001). The recombination events were generally observed to be mosaic, with donor sequences interspersed with recipient sequence. Here, we present four models to explain these observations, by fragmentation of the transformed DNA, by interruptions of the recombination mechanism, by secondary recombination of endogenous self-DNA, or by repair/replication mechanisms.

## Data Summary

Sequence data has been deposited in the European Nucleotide Archive (ENA) under accession number PRJEB27515.

**Impact Statement**

Exchange of genetic material (DNA) between bacteria is a major cause of the global spread and development of antibiotic resistance in pathogenic bacteria. Transformation, the uptake and recombination of exogenous DNA, may effectively transfer antibiotic-resistance genes or mutations between bacteria. *Neisseria meningitidis* can cause life-threatening disease in humans and is highly transformable. Here, we show for what is believed to be the first time the detailed genomic impact of experimental transformations of an antibiotic-resistance allele into the meningococcus with DNA from a same (intra) or a closely related (inter) species. Sequence alignment of the parallel transformed recipient genomes allowed for the identification of allelic replacement. The inclusion and comparison of both intraspecies and interspecies transformation, and the small antibiotic-resistance allele involving a single-nucleotide change, add to our understanding of an increasing problem. The depth of sequence coverage and cross-validated data-points ensure that the findings in the present study can reliably be added to the field of knowledge. Finally, in this study we suggest several hypotheses to explain the observed mosaic recombination patterns, increasing our understanding of horizontal gene transfer and bacterial evolution. The data is important for understanding transformation in a feared human pathogen at the genomic level for the first time.

## Introduction

In addition to replication and transcription, homologous recombination (HR) is one of the most fundamental genetic processes in nature conserved across all domains of life. The transfer of genes by horizontal gene transfer (HGT), through transduction, conjugation or transformation allows bacteria of different species to share traits (e.g. antimicrobial resistance and virulence) on a very short timescale compared to random mutation and natural selection [1].

The opportunistic pathogenic bacterium, *Neisseria meningitidis* (the meningococcus; Mc) readily takes up and incorporates extracellular DNA into its chromosome through transformation [2, 3]. In contrast to many Gram-negative bacteria, Mc is competent for transformation throughout all growth phases [4] and does not depend on induction by environmental cues (reviewed by Seitz and Blokesch [5]). Since the late 1950s, experimental transformation of the Mc with antibiotic-resistance markers has given accurate insights into the selectivity and mechanism of a highly evolved function [6, 7]. Since intraspecies transformation frequencies were early on found to be higher than interspecies transformation frequencies, preference for DNA was, prior to DNA sequencing, used for taxonomic classification of the neisseriae [4, 8]. Transformation, like sexual reproduction in general, allows for allelic re-assortment of homologous DNA [9]. The uptake and dynamic exchange of homologous DNA in competent bacteria may, through natural selection, maintain conserved regions of the genome and allow plasticity of others [10, 11]. Genomic comparisons of *Neisseria lactamica*, *Neisseria gonorrhoeae* (the gonococcus; Gc) and the Mc has shown that each species is a coherent genetic unit with more interspecies horizontal genetic exchange having taken place in the accessory than the core genome [12].

Selectivity for self-DNA in Mc and its close relative Gc is greatly influenced by preference for a specific 10–12-mer sequence, termed the DNA uptake sequence (DUS) [13, 14]. Possession of thousands of DUS copies is a genomic hallmark of the *Neisseriaceae* encompassing several dialects assorting to different species of the family [15]. Greater difference between DUS dialects severely impairs transformability. The two pathogenic *Neisseria* (Mc and Gc) and the commensal *N*. *lactamica* share the canonical 5′-ATGCCGTCTGAA-3′ DUS, facilitating their interspecies transfer. Clade-associated restriction-modification (RM) systems have also been shown to raise a barrier to transformation in Mc [16] and other bacteria [17]. Furthermore, the propensity to produce double-strand ends at unmethylated endonuclease recognition sites in endogenous DNA has been proposed to present more suitable material for HR and as such increase the rate of HR with self-DNA [18, 19]. Transformation of Mc using a heterologous marker, but not a homologous one, has been shown to depend on the number of susceptible RM sites in the donor DNA [20]. Several models for the interaction between RM systems and transformation have been proposed [21].

HR, the final step of transformation, in the Mc and Gc is dependent on the recombinase RecA, DprA and resolvases [22–25]. RecBCD has been shown to be required for efficient recombination during transformation in Gc [24]. The gonococcal RecA has been shown to possess considerably higher ATPase activity during strand displacement than that of *Escherichia coli* and found to catalyse more strand exchange through regions of microheterology [26]. At the genomic level, identification of recombination events and their endpoints in Mc and other bacteria has proven to be arduous. A particular challenge in endpoint identification is to distinguish donor-originating stretches from those that were originally present in the recipient when the sequences are identical [27, 28]. Comparing and cross-validating observed single nucleotide variants (SNVs) in a transformed recipient Mc to the donor DNA sequence enables a conservative estimate of the extent of a recombination event. Sequence divergence itself strongly influences the efficiency of HR [29–32]. Thus, acquiring sufficient resolution of sequence polymorphisms to accurately identify the recombination endpoints is a trade-off with transformation efficiency in an experimental setting.

A study of HR events by whole genome sequencing following *in vitro* transformation in *Streptococcus pneumoniae* has shown that transformation can cause fragmented or mosaic HR patterns, with multiple non-contiguous segments originating from the same molecule of donor DNA [33]. Experimental transformation of *Streptococcus pneumoniae* revealed larger recombination event sizes in the transformed recipient when allowed cell-to-cell contact with their donor, compared to *in vitro* donor DNA transformation [34]. Heteroduplex correction by mismatch repair (MMR) was not found to associate with the mosaic patterns observed. In the same study, in addition, multiple sequence imports at selected and unselected loci across the genome were frequently found. On average, 1.4 % of the recipient genome was found to have undergone recombination. Mosaic HR patterns have also been observed in a smaller fraction of *Haemophilus influenzae* transformants having used a similar protocol of genome sequencing following *in vitro* transformation [35]. The mosaicism of unselected loci was generally found within a relatively short distance from the origin of replication and, therefore, was interpreted to associate with segregation of recombination products after replication [35]. On average, the length of recombined fragments was larger in *Haemophilus influenzae* than in *Streptococcus pneumoniae* (2.3 vs 6.9 kb), but in total amounted to replacement of approximately 1 % of the genome in both species [33, 35].

Here, the genomic result of Mc transformation events was monitored by whole-genome sequencing and SNV analysis thereof. Genomic donor DNA from Mc strain FAM18 and a closely related species *N. lactamica* strain Z6793, both encoding rifampicin (rif) resistance from a single nucleotide mutation in the *rpoB* gene (Rif^R^), were used to transform Mc strain MC58 prior to whole-genome sequencing of the resulting Rif^R^ transformants. The distribution of donor-specific SNVs relative to the recipient genome was monitored using next-generation sequencing (NGS). This enabled unprecedented observations of the genetic consequences of transformation in this competent bacterium. The differences between the transformation of the two different donor DNAs and the resulting mosaic recombination events are described herein, and models to fit these observations reviewed.

## Methods

### Experimental design

**Fig. 1.**
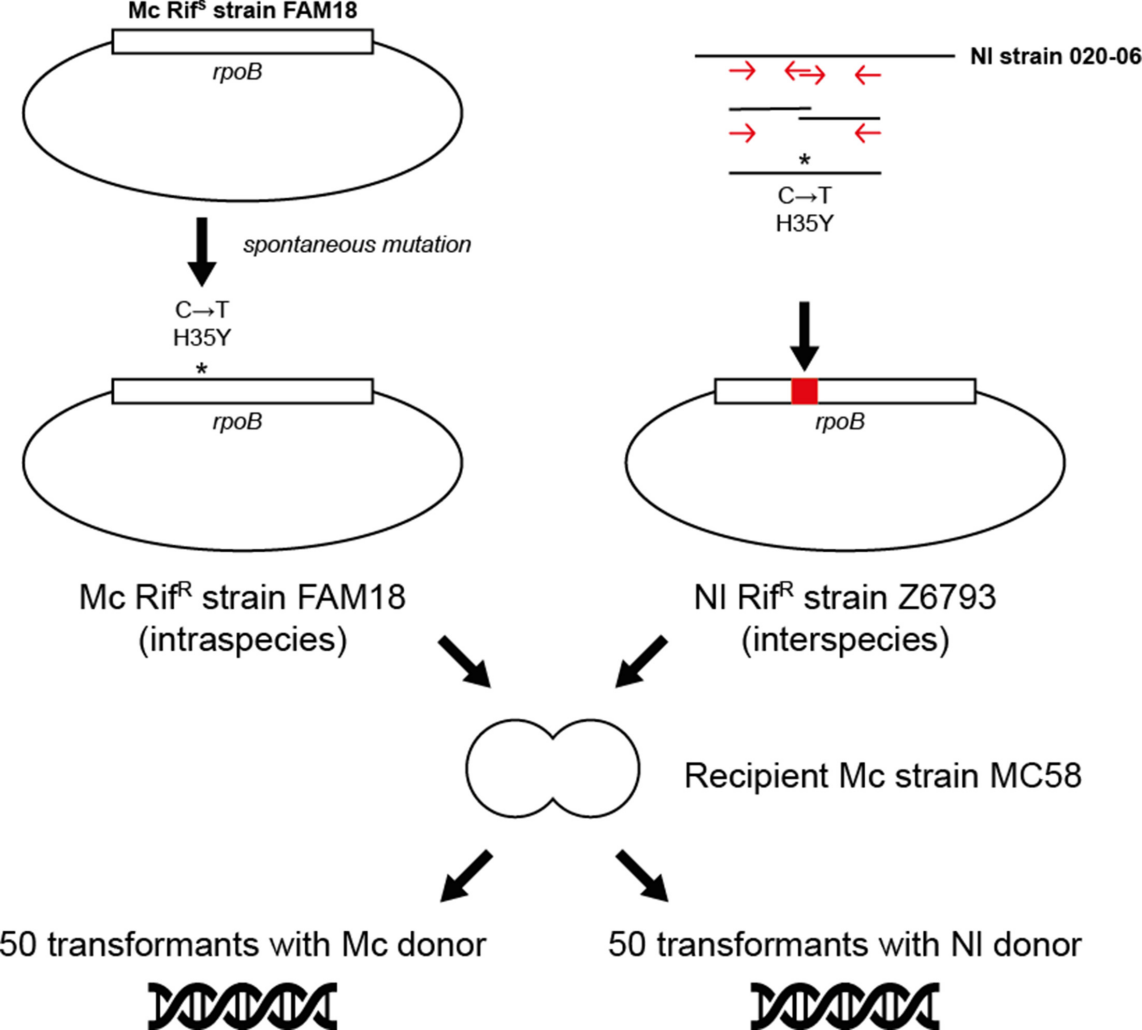
The construction and characteristics of the donor DNA, and the scheme for the intraspecies and interspecies transformation experiments. Asterisks indicate the SNV in the *rpoB* gene encoding rif resistance. The *rpoB* gene was placed within a circular chromosome (diagram not-to-scale) . Red arrows indicate the primers used to generate the RifR Z6793 donor. The recipient Mc strain MC58 is illustrated as a diplococcus. Nl, *N. lactamica*.

The well-characterized Mc strain MC58 (serogroup B) was chosen as the recipient for transformation (termed ‘MA02’) assays based on its available genome sequence and transformability (Fig. 1). The Mc strain FAM18 (serogroup C) was chosen as donor DNA (termed ‘MA01’) for its propensity to acquire rif resistance (Rif^R^) by spontaneous mutation relative to the spontaneous mutation frequency in the recipient isolate, as well as its sequence divergence from strain MC58. Thereby, the genomic impact of intraspecies transformation could be observed. The annotated genomes of both Mc strains are available in GenBank (accession numbers NC_003112.2 and NC_008767.1). In order to test interspecies transformation of MC58, the *N*. *lactamica* genomic Rif^R^ DNA had to be made first. This was achieved by transforming *N*. *lactamica* strain Z6793 with a Rif^R^ PCR product amplified using *N*. *lactamica* strain 020–06 as the template (Fig. 1, right).

### Bacterial strains and transformation assay

The Rif^R^ Mc strain FAM18 was obtained by a spontaneous single nucleotide mutation in *rpoB* with selection on gonococcal medium (GC) with rif (3 mg ml^−1^) agar plates (Fig. 1). The Rif^R^ *N*. *lactamica* Z6793 was obtained by transforming Z6793 with two combined *N*. *lactamica* Rif^R^ PCR constructs (with a total length of 412 nt). The PCR constructs were made using genomic DNA from *N*. *lactamica* isolate 020–06 as the PCR template (Fig. 1, Table S1, available with the online version of this article). Transformation of *N*. *lactamica* Z6793 with the *N*. *lactamica* Rif^R^ PCR construct followed a previously described method for spot-transformation on GC-rif plates [36]. Mutation frequencies were determined from the number of colonies on the negative control plates. The isolates used in the transformation assays are listed in Table S2. Genomic DNA was prepared from re-plated colonies following a previously described method [37]. The presence of the single nucleotide mutation in FAM18 Rif^R^ (corresponding to a H35Y change as described by Carter, Abadi, Yakubu and Pennington [38]) and the correct insertion of the PCR construct in Z6793 Rif^R^ were confirmed using PCR and sequencing using the primers listed in Table S1. In the transformation assay, the rif sensitive (Rif^S^) recipient Mc strain MC58 was transformed with the intraspecific genomic DNA of Mc FAM18 Rif^R^ or the interspecific DNA of *N*. *lactamica* Z6793 Rif^R^. Negative control experiments included genomic DNA of FAM18 Rif^S^ and Z6793 Rif^S^, as well as water – no transformants were observed in these. Single colonies of the recipient MC58 were incubated in 0.5 ml GC medium for 45 min with 2 µg genomic donor DNA ml^−1^; 50 parallel transformation assays were set up for both the intraspecies and interspecies assays. Previous tests in the laboratory have confirmed a 2 µg ml^−1^ final concentration of donor DNA to be sufficient for complete saturation of the bacterial suspensions [13]. A sample of 0.1 ml of each cell suspension was plated on GC-rif plates and incubated for 24 h. A single c.f.u. from each parallel executed assay was re-plated and incubated for another 24 h. Genomic DNA suitable for NGS was prepared from 50 Mc and 48 *N*. *lactamica* donor transformants according to a method described elsewhere [37].

### NGS DNA preparation and analysis

Two sets of sequencing libraries were prepared from the DNA extracted from each of the samples, one library using a Nextera XT DNA library preparation kit from Illumina, and a second library using KAPA HyperPlus from Roche, both following the manufacturers’ instructions. All DNA libraries were sequenced using the MiSeq sequencing platform from Illumina, the first library with 2×150 reads (v2 chemistry) and second library with 2×300 reads (v3 chemistry), following the manufacturer’s instructions. fastq reads from the first and second round of sequencing were merged for analyses in order to increase the quality and coverage (output summary in Table S2).

### Consensus sequence

A recipient consensus sequence was prepared by using a three-step method. First with a *de novo* assembly using Spades v3.10.1 [39] of the fastq reads, careful-mode enabled to reduce the number of mismatches, and insertions and deletions (indels), and coverage cut-off set to auto to reduce low coverage regions. This was followed by contig ordering of the *de novo* assembled contigs to the MC58 reference sequence (NC_003112.2) using Contiguator v2.7.5 (default settings) [40]. The resulting fasta sequence was then used as the reference in the third step, a reference alignment using Bowtie2 v2.3.0 (default setting, with the maximum fragment length, ‘maxins’, set to 600 to include large reads from the 2×300 runs) [41]. SAMtools v1.4.1 (and subscripts BCFtools and vcfutils.pl) [42] was used to convert the reference alignment output from Sequence Alignment Map (sam) to Binary Alignment Map (bam) and consensus calling (default settings). The resulting MC58 recipient consensus sequence (MA02) was henceforth used as a reference for all the transformed samples.

### SNV calling

Sequences from the transformants in fastq format were first trimmed using Trimmomatic 0.36 (removing specified adapters with setting 2 : 30 : 10; leading/trailing, 3; sliding window, 3 : 15; and minimum length, 36) [43]. This step was omitted in the three-step method for recipient consensus sequence construction to reduce loss, whereas it was found to improve the quality of the reference alignment of the samples prior to SNV calling. Sequences were then aligned to the recipient consensus sequence (MA02) using Bowtie v2.3.0 (default settings, maxins 600). SNVs were called using SAMtools v1.4.1 and VarScan v2.3 (default settings, with strand-filter disabled) [44]. In order to observe SNVs only resulting from HR following transformation of the donor DNA, a strict sequence monitoring scheme was adopted (Fig. S1). Genome-wide SNVs were identified using a stringent cut-off set to 95 % frequency when calling the donor consensus sequences to avoid potential erroneous SNVs (Fig. S1a). Therefore, SNVs in the transformants not found in the respective donors (Fig. S1b) and SNVs in the transformants not matching the donor nucleotide variant (Fig. S1c) were removed. The donor-matching SNVs were further filtered using a cut-off set to 85 % frequency following assembly to remove ambiguous bases (Fig. S1d).

### Statistical analyses and visualization

All analyses were performed using R v3.4.3 with the packages ggplot2 v2.2.1 [45] and tidyr v0.8.0 (developed by Hadley Wickham) and mass v7.3–50 [46] – the latter used to estimate shape parameters to check size distributions. Median values and empirical 95 % confidence intervals (CIs) extracted from bootstrapped intervals (2000 iterations) (with the ‘boot’ package) reported for nonparametric distributions. *Recombined regions* are stretches of sequences where all possible SNVs are in the donor variant, while interspersed regions lacking these donor SNVs were identified as *non-recombined regions*. The *recombination event* lengths are defined as the maximum distance between Rif^R^-associated SNVs in the recipients. Sequences were aligned between recipient MA02 and donors MA01 and ‘NlacRif’ of the region surrounding the resistance marker (±50 kb from Rif^R^) using the progressiveMauve algorithm (default settings – Muscle v3.6) [47], where indels relative to the recipient MA02 were excluded from the alignment. The sequence alignment was annotated using Prokka v1.13 [48], followed by manual inspection of annotated genes in Geneious v11.0.3 (whereupon genes that had been split into adjacent subunits were merged). Sequence alignments were scored using the megablast algorithm for highly similar sequences in blastn v2.8.0+ [49]. Compiling the annotated genes together with the blast alignment results was carried out using the GenoPlotR v0.8.7 package in R [50]. Sequence similarities were calculated using plotcon (default score matrix EDNAFULL) [51].

## Results

The transformation frequency of MC58 with intraspecific and interspecific Rif^R^ DNA (1.2×10^−4^ and 7.3×10^−6^ c.f.u.^−1^, respectively) was found to be three orders of magnitude higher than the spontaneous mutation frequency of the recipient (6.9×10^−9^ c.f.u.^−1^; Table S2). Initial trials revealed that recipient MC58 was transformable with DNA from *N*. *lactamica* isolate Z6793, but not with the sequenced and annotated *N*. *lactamica* 020–06. The *N*. *lactamica* donor strain Z6793 was itself competent for transformation and allowed recombination of the Rif^R^ PCR product (mean 1.2×10^−4^ c.f.u.^−1^) in the preparation of interspecific *N*. *lactamica* donor DNA. A trial to spontaneously generate Rif^R^ in the *N*. *lactamica* isolate Z6793 was not successful (Table S2). Both intraspecific and interspecific chromosomal donor DNA were extracted using the same method; gel electrophoresis revealed a large and indistinguishable fragment size distribution. Transformants of both donor DNA were phenotypically similar in size, coloration and growth rate. Forty-eight Rif^R^ isolates generated in the interspecies transformations and fifty Rif^R^ isolates generated in the intraspecies transformations were successfully prepared into genomic libraries.

The analysis of the sequencing reads was designed to ensure that only SNVs representing the donor DNA were included in the study. Briefly, assembly of *de novo* contigs to the reference sequence of MC58 was used to produce an intermediate reference sequence, the intermediate sequence was subsequently used for a final reference assembly resulting in the consensus recipient sequence henceforth used. The sequence reads of the donors and transformants were mapped against the consensus recipient sequence, and the resulting alignment was filtered in order to map only the transformants’ SNVs that matched with the donor’s SNVs. All samples were prepared and sequenced twice in order to increase the coverage and quality of the assemblies. Sufficient read coverage (>30×) following the reference-based assembly prior to SNV calling was observed for both donor and recipient sequences, as well as for the transformants (with a single exception – sample ‘M1’ with 28× mean coverage; Table S2).

**Fig. 2.**
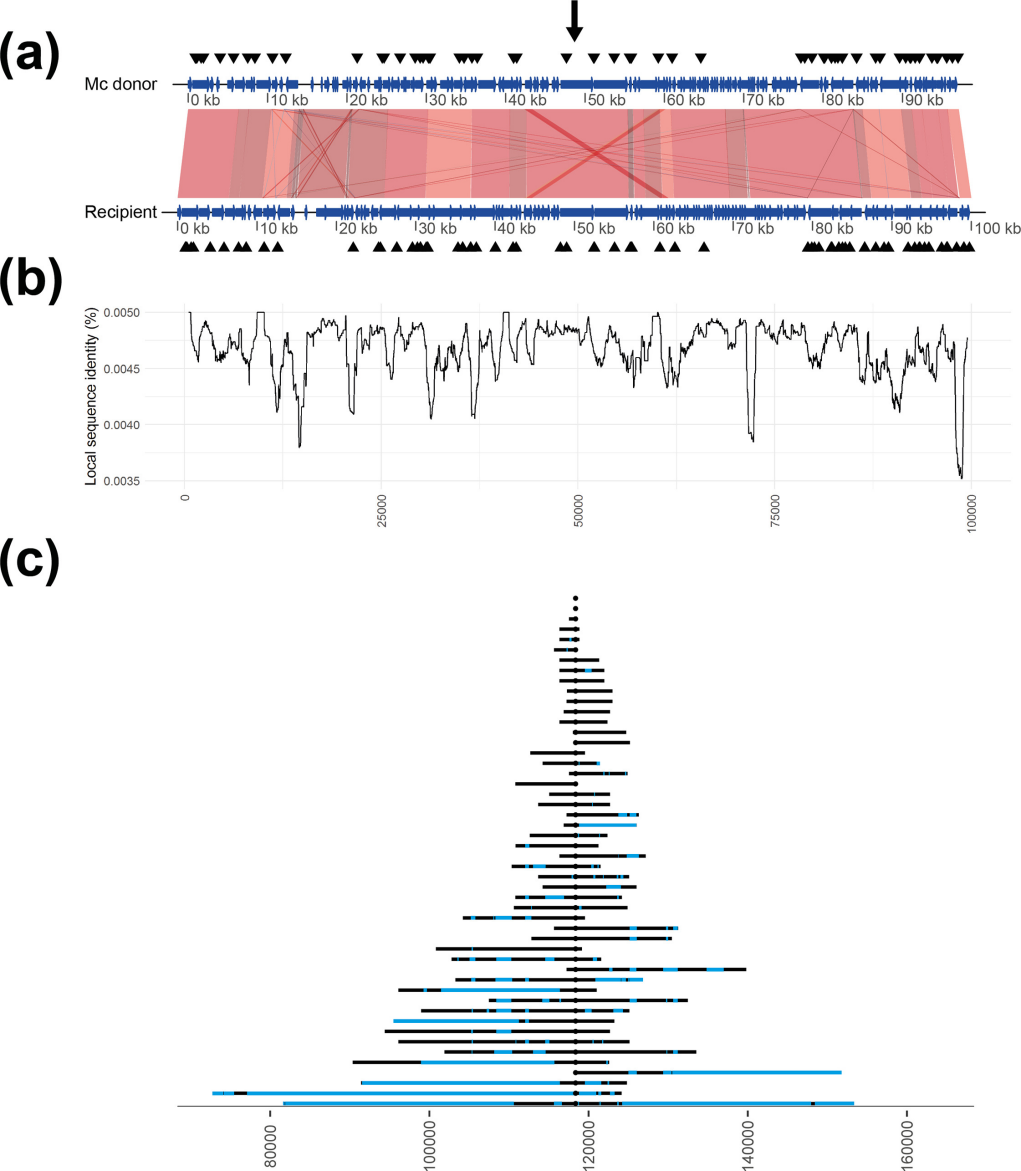
Comparative overview of donor and recipient genomes, and the results of intraspecies transformations. (a) The aligned sequences of the recipient and the Mc donor (MA01), where a gradient from grey to dark red indicates increased alignment score; annotations are shown as blue arrows and the positions of the 10-mer DUSs are shown as black triangles. The black arrow corresponds to the Rif^R^ position in the recipient (at 118 375 in our consensus sequence). (b) The sequence similarity between the recipient and the Mc donor. Positions on the *x*-axis are as in (a). (c) The maximum extent of the recombination for each sample in the experiment (the recombination event endpoints), where blue regions represent missing donor SNVs (non-recombined regions). Positions on the *x*-axis corresponds to the consensus sequence, Rif^R^ is positioned in the centre (at 118 375 nt). Each recombination event endpoint is represented by an acquired donor SNV; however, due to the scale of the figure this (these) may not be visible.

**Fig. 3.**
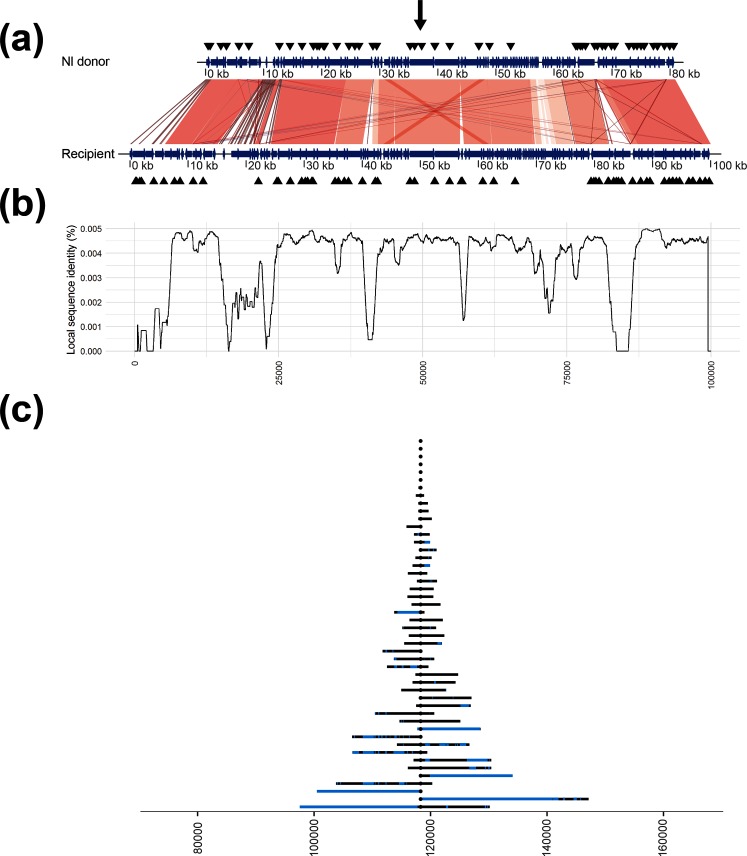
Comparative overview of donor and recipient genomes and the results of interspecies transformations. (a) The aligned sequences of the recipient and the *N*. *lactamica* donor (NlacRif), where a gradient from grey to dark red indicates increased alignment score; annotations are shown as blue arrows and the positions of the 10-mer DUSs are shown as black triangles. The black arrow corresponds to the Rif^R^ position in the recipient (at 118 375 in our consensus sequence). (b) The sequence similarity between the recipient and the *N*. *lactamica* donor. Positions on the *x*-axis are as in (a). (c) The maximum extent of the recombination for each sample in the experiment (the recombination event endpoints), where blue regions represent missing donor SNVs (non-recombined regions). Positions on the *x*-axis correspond to the consensus sequence, Rif^R^ is positioned in the centre (at 118 375 nt). Each recombination event endpoint is represented by an acquired donor SNV; however, due to the scale of the figure this (these) may not be visible. Nl, *N. lactamica*.

Recombination endpoints could hypothetically be anywhere from ±1 nt position between the last observed donor SNV and the non-observed donor SNV. A conservative approach was adopted in this study assigning only SNVs present in the donor consensus as confirmed recombined regions. Comparison of recipient and donor genomes revealed a few duplicated regions in the recipient, most importantly a 245 nt region 3.6 kb upstream and a 254 nt region 1.8 kb downstream of Rif^R^ corresponding to the homologous genes *tufA* and *tufB* (Figs 2a and 3a). A few additional, smaller duplications were identified further away (>30 kb) from Rif^R^, and a few small regions with reversed direction were found when comparing both donors to the recipient (amounting to a total of 582 and 609 nt in the 100 kb window, for the Mc and *N*. *lactamica* donors, respectively; Figs 2a and 3a). Duplicated regions may interfere with the reference sequence alignment if the size of the regions exceeds that of the sequence read length, the longer read lengths in this study (2×300) are likely long enough to bridge these duplicated regions overcoming this potential source of error.

SNVs outside the 100 kb region surrounding the Rif^R^ (position 118 375 in the recipient consensus sequence) in the Mc donor transformants included: two closely positioned SNVs found in three transformants (M14, M23 and M32) at 9.8 kb (108 kb upstream of Rif^R^), and two closely positioned SNVs found in one transformant (M1) at 50 kb (68 kb upstream of Rif^R^), plus an additional six closely positioned SNVs found in the same transformant (M1) at 1.6 Mb (1.5 Mb downstream of Rif^R^) (Table S3). For the *N*. *lactamica* donor transformants, such removed SNVs included only a single SNV found in one transformant (L28) at 1.1 Mb (1 Mb downstream of Rif^R^) (Table S3).

**Fig. 4.**
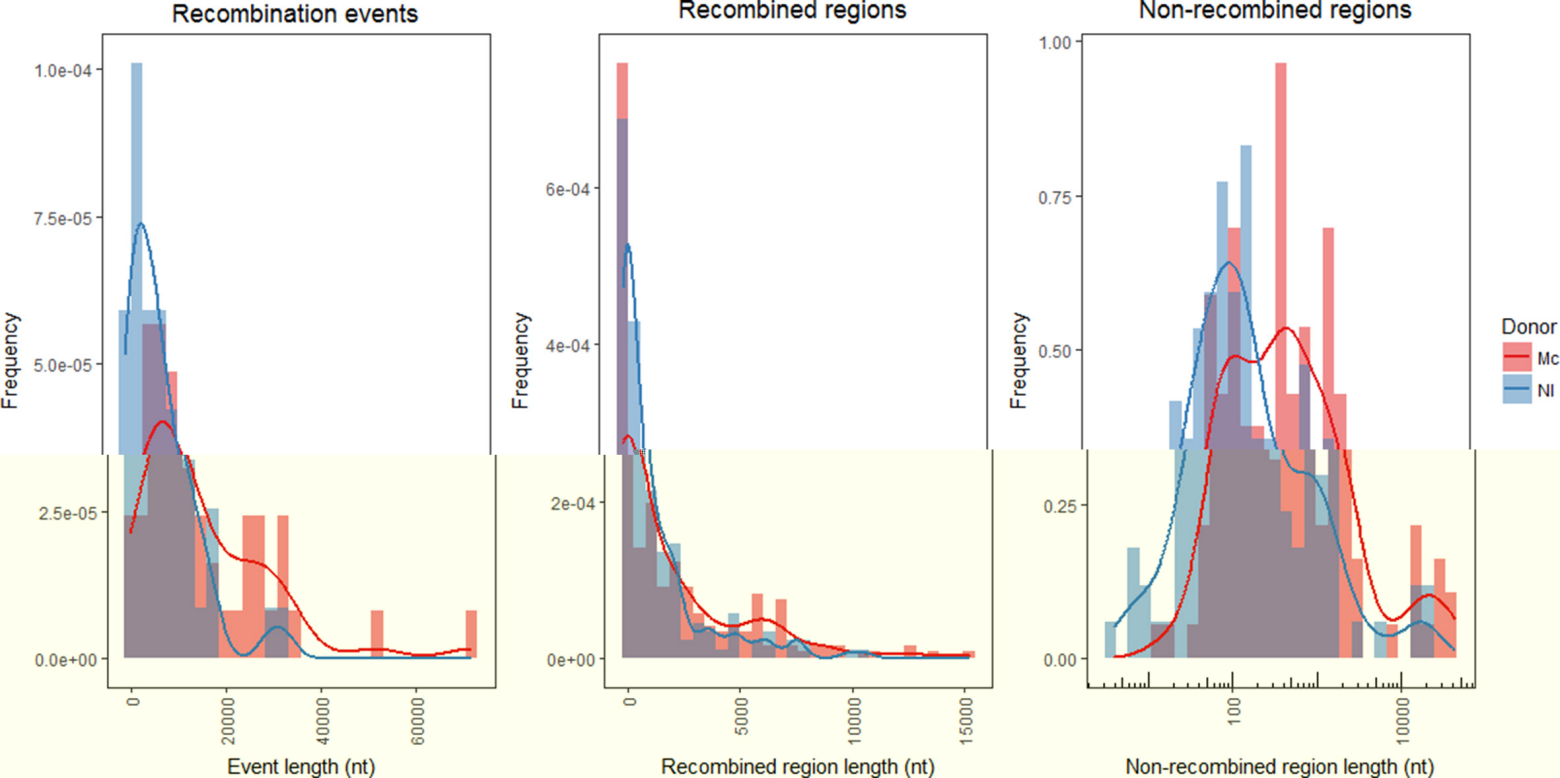
Distribution of recombination events (left), recombined regions (middle) and non-recombined regions (right) sizes for the transformants of the intraspecies (red) and interspecies (blue) donor DNA. Lines represent smoothed kernel density estimates of the histograms. Nl, *N. lactamica*.

The recombination event lengths were exponentially distributed (one-sample Kolmogorov–Smirnov test with estimated exponential shape parameter, *P*=0.12 and *P*=0.66 for the Mc and *N*. *lactamica* donors, respectively; Figs 2c and 3c). The median recombination event lengths were found to be 10 620 nt (95 % CI 6254–22 578 nt) and 5750 nt (95 % CI 6845–20 682 nt) with intraspecific and interspecific DNA, respectively. Recombination event lengths for the transformants with the Mc donor were found to be significantly longer than with the *N*. *lactamica* donor (Wilcoxon rank sum test, *P*<0.001; Fig. 4, left panel). Two of the Mc transformants and five of the *N*. *lactamica* transformants contained an unaccompanied Rif^R^ SNV without surrounding SNVs of donor origin and were hence indistinguishable from spontaneous Rif^R^ mutants (Figs 2c and 3c). The median size of the recombined regions was found to be 867 nt (95 % CI 175–1695 nt) and 505 nt (95 % CI 308–998 nt) in the intraspecies and interspecies transformations, respectively, and not significantly different from each other (Wilcoxon rank sum test, *P*=0.58; Fig. 4, middle panel). The median size of the non-recombined regions was found to be 436 nt (95 % CI 198–700 nt) and 137 nt (95 % CI 87–215 nt) in the intraspecies and interspecies transformations, respectively, and was found to be significantly longer in the intraspecies transformants (Wilcoxon rank sum test, *P*<0.001; Fig. 4, right panel). No significant difference was observed when comparing the proportion of non-recombined regions to recombined regions between the two donors (Wilcoxon rank sum test, *P*=0.53; Fig. S2). The median numbers of non-recombined regions per sample were three (95 % CI 1–5) and two (95 % CI 1–5.5) in the intraspecies and interspecies transformations, respectively, and not found to be significantly different from each other (Wilcoxon rank sum test, *P*>0.05).

**Fig. 5.**
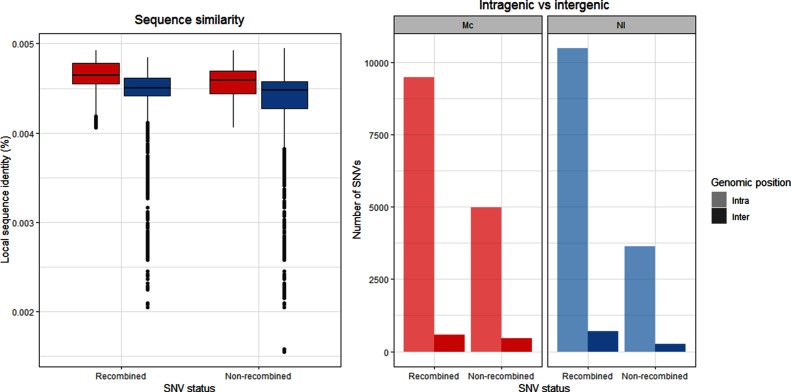
Sequence similarity of the SNVs in recombined regions and non-recombined regions (left), and the genomic position (intragenic/intergenic) of the SNVs in recombined regions and non-recombined regions (right) for the transformants of the Mc (red) and *N*. *lactamica* (blue) donor DNA. Nl, *N*. *lactamica*.

Regions of lower sequence similarity (Figs 2b and 3b) did not correspond with recombination event endpoints (Figs 2c and 3c). However, sequence similarity was found to be significantly higher in recombined regions compared to non-recombined regions for both donors (Wilcoxon rank sum test, both *P*<0.001; Fig. 5). In all transformants, the majority of all possible SNVs were found in intragenic regions (14 469 vs 1035 and 14 112 vs 951, intragenic vs intergenic positions for the transformants obtained using Mc and *N*. *lactamica* donor DNA, respectively). Accordingly, the majority of the SNVs were found in intragenic regions in the donor DNA (93.3 and 93.7 % in the Mc and *N*. *lactamica* donors, respectively). The majority of the intragenic SNVs were found in recombined regions irrespective of donor (65.6 and 74.3 % for the Mc and *N*. *lactamica* donors, respectively; Fig. 5). The number of intergenic SNVs was found to be nearly identical for both recombined and non-recombined SNVs having used the Mc donor (580 to 455, respectively), whereas a slightly higher number of recombined SNVs compared to non-recombined SNVs was found in these regions having used the *N*. *lactamica* donor (691 to 260, respectively; Fig. 5). Comparison of the variables revealed dependency of recombined versus non-recombined SNVs and position in the genome (intragenic vs intergenic) for the transformants obtained with Mc donor DNA (Pearson’s Chi-squared test, *X*^2^=39.05, *P*<0.001), but they were independent in the transformants obtained with *N*. *lactamica* donor DNA (Pearson’s Chi-squared test, *X*^2^=1.27, *P*=0.26).

## Discussion

This experimental set-up was designed to facilitate transformation of an opportunistic pathogenic bacterium to antibiotic resistance through HR of intraspecific and interspecific donor DNA. HR is known to be of great importance for the evolutionary success of competent bacteria such as *N. meningitidis*; here, we enabled a view of the genomic impact of HR by NGS with whole-genome coverage and resolution.

For highly recombinant bacteria, it is advisable to use *de novo* assemblies to account for any intrachromosomal HR events. We employed a three-step method to prepare the recipient consensus sequence, using a combination of *de novo* assembly of the reads, followed by reference-based assembly of contigs and of reads. Mapping contigs against a reference sequence, and subsequent reference-based assembly of the reads against this scaffold, allowed gaps between the *de novo* contigs to be filled. Using this consensus sequence to map all SNVs, facilitated detection of the positions of recombination events in the donors and transformants, and allowed filtering out SNVs that were not found in the donor DNA. Strict criteria, i.e. the exclusion of non-donor SNVs and ambiguous bases following assembly, were employed in order to remove SNVs that could not be assigned to HR of the donor DNA. The excluded non-donor SNVs and SNVs with ambiguous frequencies may represent HR and shuffling of self-DNA and/or a combination of self-DNA and donor DNA. The diploid genome in the Mc may allow for frequent shuffling, gene conversion and HR of heterologous self-DNA [52, 53]. Other causes for such false-positive SNVs may be spontaneous mutations or other error-prone mechanisms, such as replication slippage, antigenic variation or even sequencing errors. Indels have been shown to be incorporated during transformation and HR at low rates [54], and only maintained in a heteroduplex with the presence of matching sequences upstream and downstream [33]. Additionally, due to the complexity involved in mapping and anchoring indels relative to the recipient consensus, indels were not analysed in the present study. A similar scheme of (cross-) validation of SNVs following HR was employed by Mell, Shumilina, Hall and Redfield [55]. Croucher *et al.* [33] calculated recombined region length, median length (‘L50’), using the middle position in the region between the last observed donor SNV and the non-observed (expected) donor SNV. These regions (referred to as *boundary regions*) were found to be very variable in length in the present study. Therefore, calling the median was not considered informative here and a conservative estimate using the last observed SNVs was used instead.

The experimental transformations of live bacteria in this study generated recombination of donor DNA corresponding to a single region surrounding the Rif^R^, the selected rif-resistance allele, with very few exceptions. It is possible that despite the strict cut-off criteria and SNV validation, SNVs found outside of the Rif^R^ region in these few samples were considered false positives. These SNVs could also represent genuine recombination events; however, given the small number of clustered SNVs in these cases this seemed less likely. The few SNVs (14 and 1 with the Mc and *N*. *lactamica* donors, respectively; Table S3) found outside the 100 kb region were therefore excluded from further analyses. A similar pattern of SNVs found in high density around a selective marker has been shown for pneumococcal transformation, suggested to be the result of HR of a single donor molecule [33].

A three orders of magnitude difference in transformation frequency (efficiency) and mutation frequency in the MMR-proficient donor isolate MC58 was expected to be sufficient to avoid an issue with spontaneous Rif^R^ mutations in the transformation assays [56]. The interspecies *N*. *lactamica* donor has been shown to exhibit protein orthologue similarity at 94 % relative to the recipient Mc, compared to the intraspecies Mc donor shown with protein orthologue similarity of more than 98.7 % [57]. Thus, the transformed and recombined donor genes are not expected to express significant change in the recipient (i.e. growth or morphology), that is of course not including the rif resistance allele in *rpoB* – consequently selective pressures are not expected outside of the antibiotic-resistance marker.

Successful transformation of Mc with closely related species, such as *N*. *lactamica*, has been extensively studied (reviewed by Ambur [6]). The inability to transform the recipient Mc strain MC58 with chromosomal DNA from the *N*. *lactamica* strain 020–06 remains unresolved and warrants further investigation. The two strains share the same DUS (atDUS) [15], but display different RM profiles [58]. The transformation efficiency of *N. meningitidis* and the subsequent extent of HR is lower for interspecies events, due to divergent (non-homologous) DNA and absent or different DUS motifs [15, 59, 60], and/or different RM systems [16] or CRISPR organization [61]. Mc intraspecies transformation ratios of streptomycin resistance have been shown to be five times higher than *N*. *lactamica*-Mc interspecies transformations [60]. A small number of transformed isolates (two and five of the Mc and *N*. *lactamica* transformants, respectively) was observed with unaccompanied Rif^R^ SNVs, possibly resulting from spontaneous single-nucleotide mutations or caused by partial recombination or recombination of very small DNA fragments. The observed exponential increase in recombination event lengths in both intraspecies and interspecies transformations (Figs 2c and 3c) may support HR of partial or very small DNA fragments to mutation, as shown previously for small heterologous DNA fragments of both intraspecific and interspecific origin in *Acinetobacter baylyi* [62].

A wide range of recombination event lengths was observed in this study (Figs 2c and 3c), with significantly larger lengths for the transformants of the intraspecific (Mc) donor DNA compared to the interspecific (*N*. *lactamica*) donor DNA, with median event lengths of 10.6 and 5.8 kb, respectively (Fig. 4, left panel). It should be noted that a generalized conclusion of intraspecific versus interspecific transformation patterns in the genus *Neisseria* could not be made from this study, as this would require multiple comparative intraspecific and interspecific donors and recipients. Nevertheless, the study does show the variability of transformation patterns using two distinct donor sequences, and are in line with the differences in Mc-Mc and *N*. *lactamica*-Mc transformation rates found by Hoke and Vedros having used a streptomycin-resistance marker [60]. The observed range of recombination event lengths was in agreement with previous estimates in *N. meningitidis* (1.5–9.9 kb in work by Linz, Schenker, Zhu and Achtman [63]) and *Haemophilus influenzae* (1.2–16.6 kb in work by Mell *et al*. [55]), but considerably larger than estimates from *Helicobacter pylori* (1.3 kb in work by Lin *et al*. [64]). HGT by means of generalized transduction or conjugation and recombination of fragments from 40 to 107 kb, or when including mobile elements, up to 240 kb, has been suggested between closely related *E. coli* clones [65]. If such lengths can be observed in the case of natural transformation, and whether exogenous DNA of such recombination substrate lengths would remain intact, remains to be determined. The wide distribution of recombination event lengths observed in this study is similar to that shown in transformation and HR in *Streptococcus pneumoniae* [33, 34], but different to the complete heterogenic distribution of recombined fragments shown in experimental HGT in *E. coli* [66]. Even though the recombination endpoints did not significantly correlate with reduced sequence similarity in this study, it is expected that overall reduced homology will limit the efficiency of transformation and HR, and likely be the cause of the significantly larger recombination events observed in the intraspecies relative to the interspecies transformations.

Fragmented integration of DNA during HR as shown in the present study (Figs 2c and 3c) has been shown previously [33–35, 55, 64]. It is possible that the length of these recombined regions is underestimated in this study because of the strict criteria calling recombined regions only from the last observed donor SNVs. The length of the non-recombined regions, however, may be overestimated as these were called in-between the last observed donor SNVs (Fig. 4, middle and right panels). The distribution of recombination events was found to be similar in transformants with both donor DNAs, whereas non-recombined regions were found to be significantly larger for the intraspecies transformants compared to the interspecies transformants. The large recombination event lengths observed in the present study may not cause epistatic interactions to be at odds with HR [67], indeed through evolutionary pressures these two mechanisms may work in synergy. The comparable proportion of non-recombined regions to recombination event lengths in both transformations indicate that the larger non-recombined regions found in intraspecific transformants were not a direct consequence of the longer observed recombination fragment lengths. Biological effects and subsequent evolutionary pressures have been suggested to explain the observation of non-recombined genes in the transformation of divergent strains of *A. baylyi* [68], the mechanisms involved in how specific genes would avoid HR, and the absence of HR outside a delimited region shown in this study, remain to be addressed. In the present study, non-recombined regions were not found to be more likely in intergenic regions; however, care must be taken since the intergenic region makes up far fewer SNVs than the intragenic regions (Fig. 5, right panel).

**Fig. 6.**
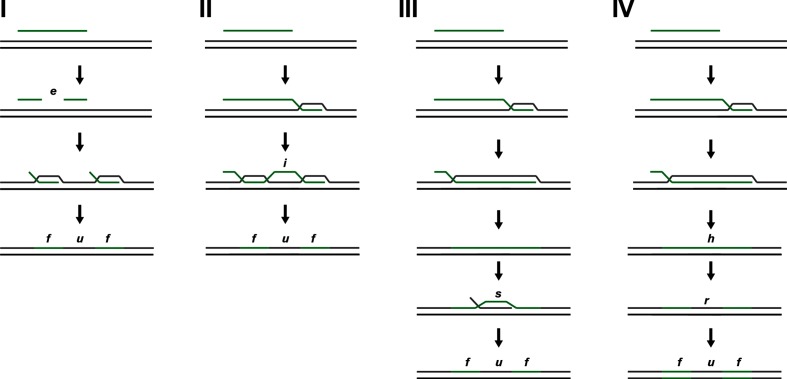
Models of HR following transformation in *N. meningitidis*. Model I shows fragmentation (e.g. by endonuclease digestion of DNA - *e*) of the transformed DNA prior to recombination, resulting in HR of two fragments (*f*) flanking a non-recombined region (*u*). Model II shows interruptions (*i*) in the recombination mechanism resulting in interspersed integration of DNA. Model III shows how secondary recombination of self-DNA (*s*) may introduce a region (*u*) without the SNVs in the original recombination pattern. Model IV shows how strand-dependent MMR (*r*) following recombination can introduce non-recombined regions (*u*) in the original recombination pattern – asynchronous heteroduplex segregation of the donor region on one strand (*h*) during DNA replication may also introduce non-recombined regions.

Here, we suggest four models for the observed mosaic patterns of recombined regions interspersed with non-recombined regions, although these may not be mutually exclusive. (I) One model for the origin of the non-recombined regions during HR is by cleavage of the donor DNA by endonucleases (i.e. the models proposed by Johnston *et al.* [21]), counteracted by the potential protection of endogenous DNA from digestion by methylation [35, 64] (Fig. 6, I). DNA is transferred across the inner membrane as ssDNA through ComA, and aided by the PilG protein and other inner-membrane components during Mc transformation [69–71], and increased Mc transformation efficiency in the absence of recipient-specific NlaIV restriction sites in plasmid donor DNA but not genomic homologous DNA has been demonstrated [20]. No correlation was shown between the position of methylation sites and recombination endpoints in *Helicobacter pylori* transformation [64]; however, certain type I endonucleases may cleave the DNA at variable positions away from the recognition sequence [72]; thus, making it difficult to assess correlations [68].

(II) A second model involves the propensity for interruptions of the recombination mechanism and fidelity itself, causing collapse of the D-loop (Fig. 6, II). Secondary structures on the DNA strand (such as transcriptional terminators, stem-loop structures) and/or stretches of non-homologous DNA or indels have been shown to reduce transformation efficiency [54]. In the present study, a significantly higher sequence similarity was found in the SNVs making up the recombined regions compared to the sequence similarity in SNVs in the non-recombined regions (Fig. 5, right panel), supporting this model; however, no correlation was found between recombined regions and sequence similarity in a genome-wide transformation assay with *Streptococcus pneumoniae* [33].

(III) A third model suggests continuous recombination of the donor strand, whereas secondary recombination events with endogenous self-DNA introduce what is interpreted to be ‘non-recombined regions’ (Fig. 6, III). Mc and its close relative Gc have been shown to be diploid (whereas other relatives, such as *N*. *lactamica*, have been found to be haploid) [52, 53). Efficient gene conversion and intracellular transfer of DNA confirmed by antigenic variation has been shown in Mc in the absence of transformation [73, 74]. As such, available self-DNA should be present and readily exchanged within the cell, supporting this model. It is difficult to find support for this model in the present study. A certain distribution and limitations to the size of the intracellular self-DNA may be expected and consequently the size of the non-recombined regions. The observed wide range of the non-recombined regions seems plausible to support secondary recombination of self-DNA, as a greater sequence similarity and consequently more efficient secondary recombination may explain the increased lengths of the apparent non-recombined regions observed between the recipient and the Mc donor compared to the *N*. *lactamica* donor (Fig. 4 , right panel).

(IV) A fourth model also suggests continuous recombination of the donor strand, where other mechanisms may influence the resulting recipient's genome. Strand-specific post-replication repair pathways (e.g. the MMR pathway) may correct a portion of the recombined donor DNA resulting in a region with self-DNA (Fig. 6, IV); however, a study of pneumococcal transformation failed to identify a role of MMR in the generation of such mosaicism [33]. Asynchronous heteroduplex segregation of donor DNA on a single strand during DNA replication may result in breaking up and shuffling of the recombined region, introducing self-DNA into the recombined regions in the daughter cells [35] (Fig. 6, IV). The reported uniform (and hence random) propensity for gene conversion of *tuf* genes in *Salmonella* [75] may similarly in the present study explain the size distribution of non-recombined regions (Fig. 4, right). The diploid nature of Mc (as mentioned in III) may provide the homologous DNA for gene conversion in the case of double strand breaks (as shown in *Salmonella enterica* [76]).

All four models rely on error-prone strand-specific DNA repair pathways (e.g. MMR), allowing for heteroduplex recombination products to arise, otherwise these would be corrected (to self-DNA) prior to DNA replication. Saturation of the MMR following transformation in both *Streptococcus pneumoniae* and *N. meningitidis* has been observed [77, 78]. The mechanisms (repair and asynchronous segregation) described in the fourth model can be engendered subsequently to any of the other hypotheses, making efforts to identify a single mechanistic explanation for this complex scenario difficult. Finally, the biological consequences (i.e. genes affected) and the varying evolutionary pressure of these may influence the actual isolates we observe following transformation and HR. Detrimental changes, complete or partial genes, or allelic changes of the transformed recipient may be severe such that it is not recovered, or it may favour those that through secondary recombination events restore the specific parts of the genome (e.g. Fig. 6, III and IV).

The present study extends previous efforts to observe and understand the mechanisms involved in HR in naturally transformable bacteria, allowing pathogens to transfer adaptive genetic elements introducing beneficial traits and other changes, such as antibiotic resistance. The combination of experimental transformation and sequencing revealed a complex pattern of both DNA transfer and a wide range of recombination event lengths – with recombined regions interspersed with non-recombined regions. The four models presented may in combination, or individually, explain the pattern of interspersed recombined and non-recombined SNVs observed. Further understanding of these mechanisms may identify targets in the bacterial life cycle that effectively neutralize the ability to successfully transfer and take up genetic elements. This would be extremely beneficial in the combat against infectious bacteria and the ongoing struggle to deal with HGT and antimicrobial resistance.

## Data bibliography

1. Alfsnes *et al*. ENA. PRJEB27515 (2018).

## Supplementary Data

Supplementary File 1
