# Supplementary material for: A genomic view of experimental intraspecies and interspecies transformation of a rifampicin-resistance allele into Neisseria meningitidis
Source: Microb Genom. 2018 Sep 25;4(11):e000222. doi: 10.1099/mgen.0.000222 (PMC6321871; doi:10.1099/mgen.0.000222)
Supplement: Supplementary File 1 [file mgen-4-222-s001.pdf]

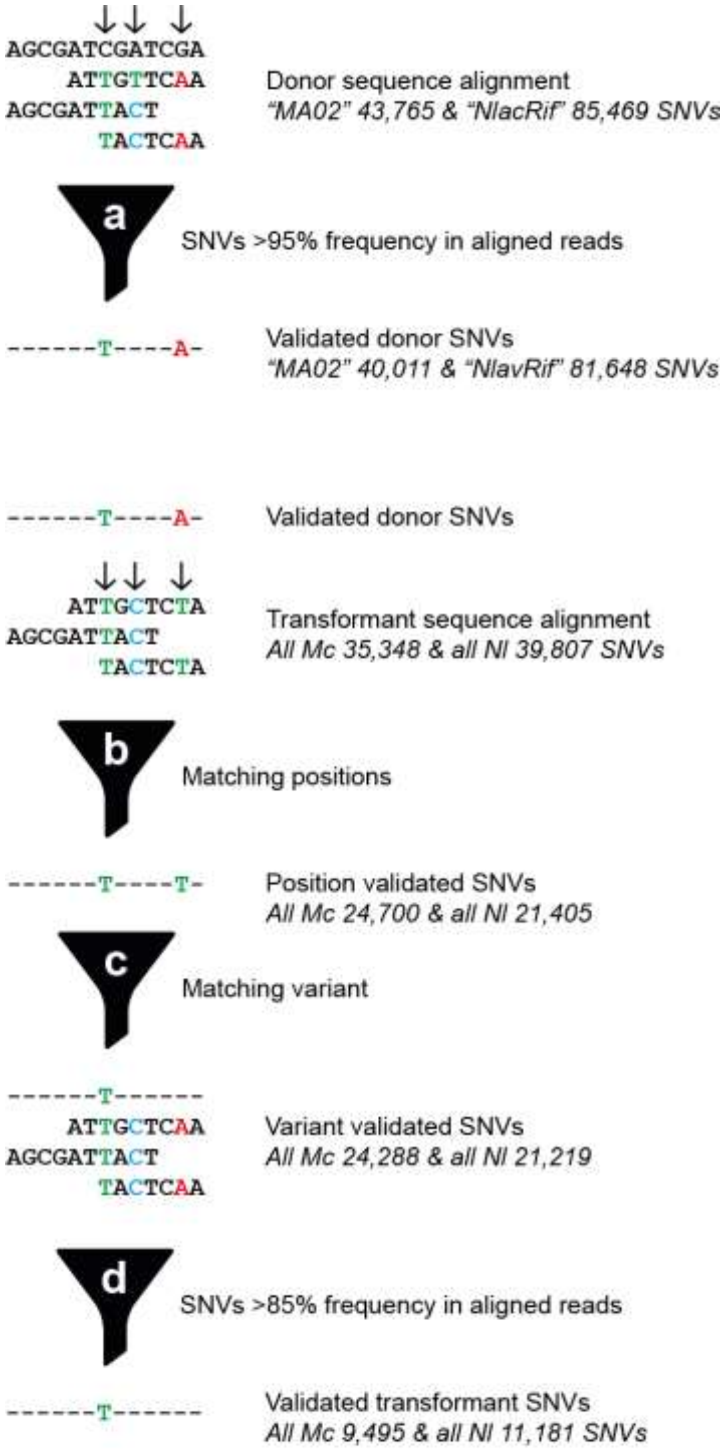

Figure S1 – Cross-validation and SNV calling scheme. Donor sequence reads were aligned against the consensus reference sequence - SNVs found presented in less than 95% of the reads were excluded (a). SNVs identified for each transformant sample aligned against the consensus sequence was matched to the donor validated SNVs for positions (b) and same nucleotide variant (c). Finally, the SNVs matching the donor positions and nucleotide variants were filtered by excluding SNVs found in less than 85% of the aligned reads (d). Numbers in italics indicate the number of SNVs for the two donors Mc "MA02" and Nl "NlacRif" (top), and all combined Mc and Nl transformants (bottom).

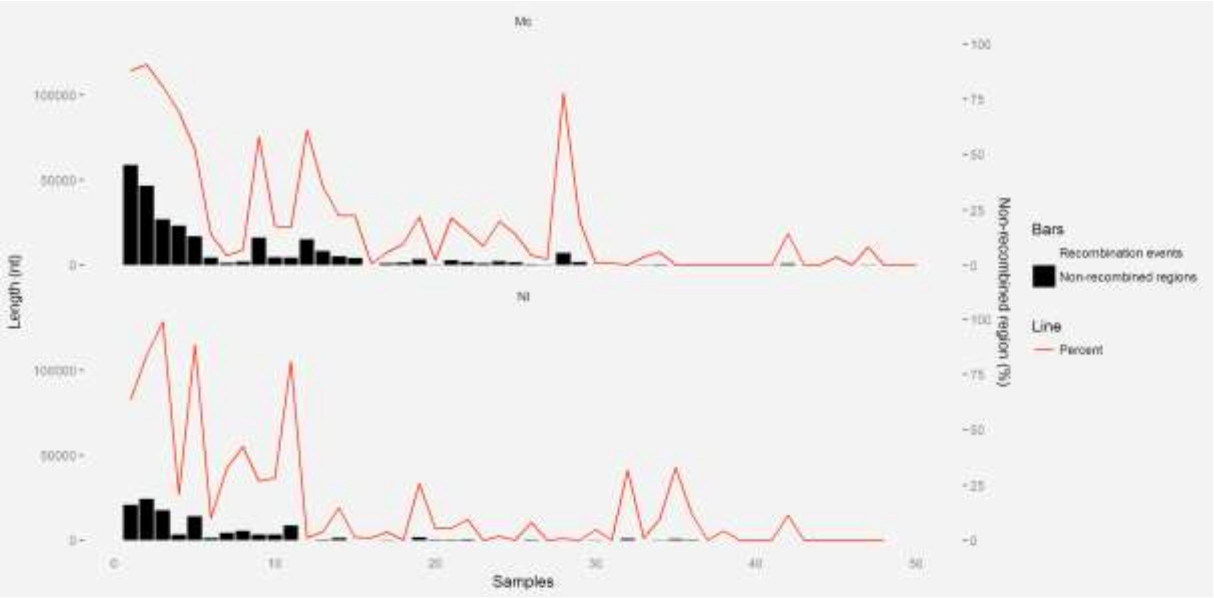

Figure S2 – Bars represent length (nt – left axis) of recombination event size and combined size of all non-recombined regions for each sample (x-axis) for the Mc donor (top) and the Nl donor (bottom), line represents percent (% - right axis) non-recombined regions out of recombination event length.

Table S1 - Primers used to check for spontaneous mutation and primers used for the 412 nt construct made using Nl isolate 020-06 as template and transformed into Nl Z6793 Rif^R (the Nl donor)

**Primers**	**Description**	**Primer sequence**	**Reference**
NmB9F/NmB24R	Mc *rpoB* mutation PCR & sequencing	GATTAATGCAAAACCTGT/ AAACGCCTGAAGGTCCGAA	*Nolte et al. 2003*
JEE40/JEE43	Nl Rif^R construct 1/2, PCR & sequencing	ATCCGAAAACTTGATGCCG/ CCCATTGTCTGAAGTAACC	*This study*
JEE41/JEE44	Nl Rif^R construct 2/2, PCR & sequencing	ATAAACGCCGTGTATCTG/ ACCGAAGAAATCGATTACTTGTC	*This study*

Table S2 - Summary of the isolates and samples included, spontaneous Rif^R mutation frequency and Rif^R transformation frequency, the number of experimental samples sequenced, number of reads and sequence coverage

	**Number of samples/parallels**	**Average spontaneous Rif^R frequency cfu^{-1}†**	**Average # of reads***	**Average coverage****
Recipient *N. meningitidis* MC58 (*model strain*)	1	6.9E-9	22273 / 353573	227
Donor *N. meningitidis* FAM18 Rif^R (*this study*)	1	3.6E-8	34509 / 172278	518
Donor *N. lactamica* Z6793 Rif^R (*this study*)	1	N/A	21309 / 581072	374
		**Average transformation frequency cfu^{-1}†**		
MC58 (recipient) + FAM18 Rif^R (Mc donor)	50	7.3E-6	34030 / 168447	225 (28-1455)
MC58 (recipient) + Z6793 Rif^R (Nl donor)	48	1.2E-4	33943 / 198375	245 (96-647)

† Average of 3 replicates – transformation set-up like described in the materials and methods. cfu = colony forming units.
* Average number of reads from the first sequencing effort using MiSeq 2x150 bp (v2) and second sequencing effort using MiSeq 2x300 bp (v3).
** Average coverage for the recipient is calculated at the third step following Spades and Contiguator assemblies, average coverage for the donor and sample sequences is shown for the sequence alignment against the recipient consensus – range of coverage for all samples shown in parenthesis. A single sample was found with <30× average coverage (sample "M01").

Table S3 – SNVs outside the 100 kb region surrounding Rif^R

**Sample name***	**Number of excluded SNVs**	**Positions in consensus sequence ("MA02")**
M1	2+6	50545, 50653; 1663244, 1663250, 1663252, 1663257, 1663258, 1663264
M14	2	9814, 9832
M23	2	9814, 9832
M32	2	9814, 9832
L28	1	1105390

* M and L represent Mc and Nl donor transformants respectively.
